# Primary tuberculosis cutis orificialis; a different face of the same coin

**DOI:** 10.1016/j.idcr.2021.e01305

**Published:** 2021-10-07

**Authors:** Gawahir.A. Ali, Wael Goravey

**Affiliations:** Department of Infectious Diseases, Communicable Diseases Centre, Hamad Medical Corporation, Doha, Qatar

**Keywords:** Tuberculosis, Cutis orificialis, Mucosa ulcer, Golimumab

## Abstract

Primary tuberculosis cutis orificialis (TCO) is a rare form of extrapulmonary TB. The Lack of respiratory symptoms and similarity of the presentations to other oral lesions can pose a diagnostic dilemma. Hence, delaying treatment and potentially devastating consequences

A 51-year-old female was referred to our service because of a three-month history of a painful and non-healing oral ulcer on the right buccal mucosa associated with a weight loss of 4 kg. Her history is significant for well-controlled seronegative rheumatoid arthritis (RA) with methotrexate and golimumab for the two years. She denied fever, painful joints, or cough. Examination of her mouth was limited by pain when she fully opened her mouth, but a single right buccal ulcer opposite to the retromolar trigone was seen (Fig. 1A). It was tender with ragged margins (around 2.1 cm). No other findings or lymphadenopathy were observed. The differential diagnoses included squamous cell carcinoma, infectious etiologies, methotrexate related, or RA related. Laboratory tests were within normal limits except for a CRP level of 40 mg/L (0−5). Anti-CCP, HIV, and syphilis serology were negative. Punch biopsy showed necrotizing granulomatous inflammation with scattered acid-fast bacilli (Fig. 2A and B). The fungal culture was negative. However, MTB (GeneXpert MTB/RIF) was positive. Subsequently, a fully sensitive MTB strain was isolated from the culture. CXR demonstrated no pulmonary involvement, and MRI of the jaw excluded osteomyelitis. She was started on standard 6 months TB therapy with pain resolution and complete healing of the ulcer (Fig. 1B). Golimumab was stopped. She had no recurrence for six months during follow-up.

**Fig. 1:**
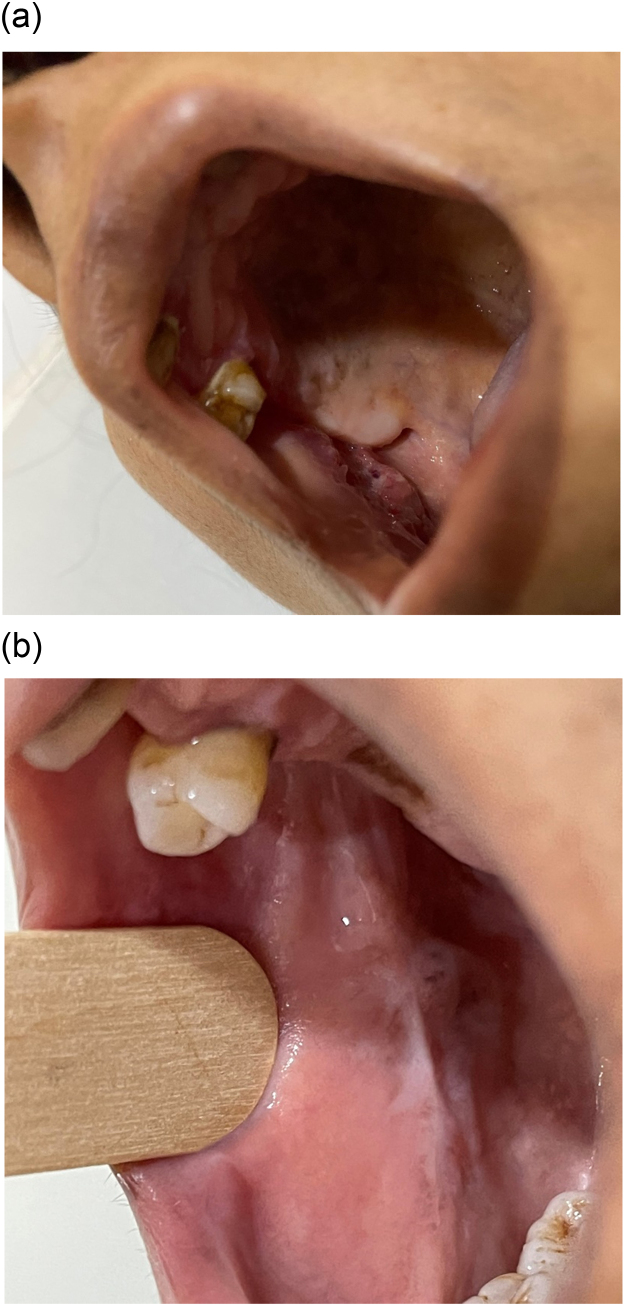
A: Single right buccal ulcer with ragged margins. B: Right buccal ulcer demonstrating significant improvement after six months of the TB therapy.

**Fig. 2:**
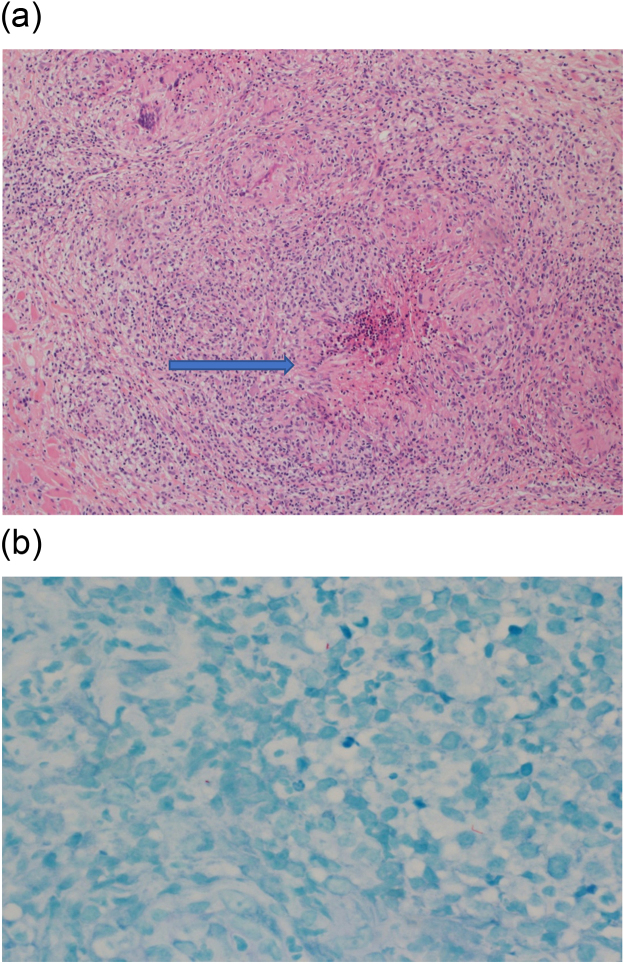
A: Punch biopsy showed necrotizing granulomatous inflammation consistent with TB (arrowed). B: Punch biopsy with Ziehl-Neelsen stain showed scattered acid-fast bacilli.

Primary TCO occurs when the oral mucosa is affected without pulmonary involvement [1]. It represents 0.01–5% of all TB cases [1]. Children and adolescents are affected more than adults, while tongue and palate are the most common sites for infection [2]. Various morphological lesions exist including ulcers, nodules, plaques, and fissures [3]. It can cause osteomyelitis in the adjacent bone [1]. Typically, primary TCO is a single and painless lesion. The differential diagnoses are squamous cell carcinoma, chronic traumatic ulcer, medications, syphilis, and other granulomatosis diseases [3]. Notably, golimumab at least doubles the risk for TB activation [4].

The first step to clinch the diagnosis is to consider TCO while evaluating any chronic oral lesions [1]. Fundamentally, TB MTB PCR can secure the diagnosis promptly pending the histopathological diagnosis to rule out other potential causes [1]. The mainstay of management is 6 months of TB therapy [3].

## Funding

No funding was received towards the publication.

## Author contributions

**GA:** Clinical management, data acquisition and manuscript writing. **WG:** Clinical management, contribute to data acquisition, manuscript preparation and final proof reading.
